# Attenuation of indomethacin-induced gastric mucosal injury by prophylactic administration of sake yeast-derived thioredoxin

**DOI:** 10.1007/s00535-012-0564-5

**Published:** 2012-03-09

**Authors:** Atsushi Nakajima, Toshiro Fukui, Yu Takahashi, Masanobu Kishimoto, Masao Yamashina, Shinji Nakayama, Yutaku Sakaguchi, Katsunori Yoshida, Kazushige Uchida, Akiyoshi Nishio, Junji Yodoi, Kazuichi Okazaki

**Affiliations:** 1 Division of Gastroenterology and Hepatology, The Third Department of Internal Medicine, Kansai Medical University, 10-15 Fumizono-cho, Moriguchi, Osaka 570-8506 Japan; 2 Department of Biological Responses, Institute for Virus Research, Kyoto University, Kyoto, Japan; 3 Center for Cell Signaling Research/CCSR and Department of Bioinspired Sciences, Ewha Womans University, Seoul, Republic of Korea

**Keywords:** Thioredoxin, NSAIDs, Indomethacin, Gastric mucosal injury, Sake yeast

## Abstract

**Background:**

Indomethacin is one of the group of nonsteroidal anti-inflammatory drugs, which often cause gastric mucosal injury as a side effect. Infiltration and activation of inflammatory cells, production of proinflammatory cytokines and chemokines, generation of reactive oxygen species, and activation of apoptotic signaling are involved in the pathogenesis of indomethacin-induced gastric injury. We examined whether sake yeast-derived thioredoxin (a small redox-active protein with anti-oxidative activity and various redox-regulating functions) reduced indomethacin-induced gastric injury.

**Methods:**

Gastric injury was produced by the intraperitoneal administration of indomethacin (40 mg/kg body weight) to C57BL/6 mice. Prior to the administration of indomethacin, the mice were offered food pellets containing non-genetically modified sake yeast-derived thioredoxin (thioredoxin 200 μg/g) for 3 days. Histological examinations, assessment of myeloperoxidase activity, and analysis of the gene expressions of proinflammatory cytokines and a chemokine (interleukin [IL]-1β, IL-6, and CXCL1) were statistically evaluated. Indomethacin cytotoxicity was determined by lactate dehydrogenase release from murine gastric epithelial GSM06 cells induced by 24-h treatment with 200 and 400 μM indomethacin after 1-h preincubation with 100 μg/ml sake yeast-derived thioredoxin.

**Results:**

Macroscopic (edema, hemorrhage, and ulcers) and histological (necrosis, submucosal edema, neutrophil infiltration) findings induced by indomethacin were significantly reduced by pretreatment with food pellets containing thioredoxin. Gastric myeloperoxidase activity and the gene expressions of proinflammatory cytokines (IL-1β and IL-6) were also significantly reduced by this pretreatment compared with findings in the mice not pretreated with thioredoxin-containing food pellets. The administration of sake yeast-derived thioredoxin significantly reduced indomethacin-induced cytotoxicity in GSM06 cells.

**Conclusions:**

We conclude that oral administration of sake yeast-derived thioredoxin reduces indomethacin-induced gastric injury. Sake yeast-derived thioredoxin may have therapeutic potential against indomethacin-induced gastric injury.

## Introduction

Nonsteroidal anti-inflammatory drugs (NSAIDs) such as indomethacin are widely used as anti-inflammatory and analgesic agents. However, gastrointestinal injury is a serious adverse effect of NSAIDs, and effective strategies are required to protect the gastrointestinal mucosa. Many previous studies have investigated the mechanisms of the development of NSAID-induced gastric mucosal lesions [1–3]. NSAIDs may cause gastric lesions by inhibiting cyclooxygenase (COX) and reducing prostaglandin (PG) production [4], but the exact pathogenic mechanism remains to be elucidated. Several investigators have reported that the intraperitoneal injection of anti-neutrophil serum or the immunoneutralization of adhesion molecules on neutrophils and endothelial cells significantly attenuates the gastric mucosal injury induced by NSAIDs [5, 6]. Therefore, neutrophil activation and infiltration into the stomach appear to contribute to the gastric mucosal lesions induced by NSAIDs.

Oxidative stress induced by reactive oxygen species (ROS) has an important role in the gastric mucosal injuries induced by various stressors, including NSAID administration [7]. There are some possible sources of ROS in NSAID-induced gastric injury, such as infiltrating inflammatory cells (particularly neutrophils) and gastric epithelial cells. The redox state is finely tuned to preserve cellular homeostasis through the expression and regulation of oxidant and antioxidant enzymes. Mammalian cells have a complex network of antioxidants, such as catalase, superoxide dismutase (SOD), and glutathione peroxidase to scavenge ROS. In addition to these enzymes, the members of a family of thiol-disulfide oxidoreductases act as cytoprotective antioxidants. Glutathione and thioredoxin (TRX) are the two most important redox-regulating molecules considered to regulate various cell functions such as cell growth, apoptosis, and cytoprotection [8]. TRX is a small multifunctional protein that contains a redox-active disulfide/dithiol within the conserved active site sequence(-Cys-Gly-Pro-Cys-). TRX is induced by various types of stresses and protects cells from such stresses, including viral infection, exposure to ultraviolet light, X-ray irradiation, and hydrogen peroxide (H_2_O_2_) [9]. Moreover, TRX scavenges ROS such as singlet oxygen, hydroxyl radicals, and H_2_O_2_ [10]. Previous studies have demonstrated that the overexpression of TRX in transgenic mice or the administration of recombinant human TRX attenuated focal ischemic brain damage [11], adriamycin-induced cardiotoxicity [12], thioacetamide- or lipopolysaccharide-induced acute hepatitis [13], *Helicobacter felis*-induced gastritis [14], cerulein- and lipopolysaccharide-induced chronic pancreatitis [15], dextran sulfate sodium (DSS)-induced colitis and colonic inflammation in interleukin (IL)-10 knockout mice [16], and indomethacin-induced gastric mucosal injury [17]. It remains unclear, however, whether non-genetically modified TRX, which is efficiently extracted from *Saccharomyces cerevisiae* (*S. cerevisiae*, Japanese sake yeast cells), can attenuate indomethacin-induced gastric injury.

In the present study, we investigated the prophylactic efficacy of orally administered sake yeast-derived TRX against indomethacin-induced gastric mucosal injury.

## Methods

### Extraction of TRX from *S. cerevisiae* and composition of food pellets

As previously reported [18], TRX is efficiently extracted from Japanese sake yeast cells (*S*. *cerevisiae*) following treatment with ethanol and heat stresses. Initially, specially treated Japanese sake (Japanese rice wine), which contained a high level of TRX, was lyophilized after removing the ethanol with an evaporator in vacuo, and the dried material included 2000 μg/g of TRX. Commercial mouse food pellets (CE-2; Clea Japan, Tokyo, Japan) were mixed with 10% of the dried TRX material (Clea Japan; TRX content: 200 μg/g). As a reference, normally treated Japanese sake, which contained very little TRX, was lyophilized by the same procedure. Control food pellets contained the same dried material components, except for TRX.

The lyophilized material that contained a high (or low) level of sake yeast-derived TRX was kindly provided by Redox Bioscience (Kyoto, Japan) in cooperation with Kizakura (Kyoto, Japan).

### Mice and prophylactic administration of TRX-containing food pellets

Female C57BL/6 mice at 8 weeks of age, weighing around 20 g, were purchased from Japan SLC (Shizuoka, Japan). All mice were bred at the animal facility of Kansai Medical University under specific-pathogen-free conditions and were kept in cages in a temperature- and humidity-controlled room with a 12-h dark-light cycle throughout the experiment. Prior to the administration of indomethacin, the mice were offered the custom-manufactured food pellets described above and tap water for 3 days.

The Ethics Committee for the Use of Experimental Animals of Kansai Medical University approved all experimental protocols.

### Indomethacin-induced gastric mucosal injury model

Indomethacin-induced gastric injury was produced by the intraperitoneal administration of indomethacin (800 μg/body, 40 mg/kg body weight). Indomethacin was dissolved in physiological saline (1000 μg/ml), and 0.8 ml of the solution was administered. The control mice were administered the vehicle (physiological saline) intraperitoneally. After 24 h of starvation, the mice were killed under pentobarbital sodium anesthesia by cervical dislocation. Their stomachs were removed, opened along the lesser curvature, and washed with phosphate-buffered saline (PBS). Two blinded observers examined the extent of gastric mucosal lesions macroscopically.

Thereafter, the mice were divided into the following 4 groups, according to the presence or absence of the administration of indomethacin and TRX-containing food pellets: (1) administration of indomethacin and control food pellets (Indomethacin-Control), (2) administration of indomethacin and TRX-containing food pellets (Indomethacin-TRX), (3) administration of physiological saline and control food pellets (Saline-Control), and (4) administration of physiological saline and TRX-containing food pellets (Saline-TRX).

### Histological analysis of indomethacin-induced gastric mucosal injury

After fixation with 4% phosphate-buffered formaldehyde (pH 7.2) and embedding in paraffin, the stomachs were cut with the long axes parallel from the fundus to the antrum, perpendicular to the mucosal surface, and the sections were stained with hematoxylin–eosin. Two experienced investigators, who were blind to the group of mice, performed histological examinations of coded slides regarding epithelial damage (necrosis), thickness of the submucosal layer (edema), and degree of neutrophil infiltration, as follows. Epithelial damage was defined as necrosis (and apoptosis) of the epithelium and the disappearance of luminal structures. The assessment involved determining the percentage of (antral) epithelial damage, which was calculated as follows:

$$ \begin{gathered} {\hbox{ Percentage }}( \%){\text{ of epithelial damage}} = \left\{ {{\text{length of }}\left( {\text{antral}} \right){\text{ lesion/whole length }}} \right. \hfill \\ \left. {{\text{of }}\left( {\text{antral}} \right){\text{ epithelium}}} \right\} \times 100 \hfill \\ \end{gathered} $$

Three well-oriented and perpendicularly sliced (antral) gastric wall portions were selected for measuring the thickness of the submucosal layer (edema), and the results were averaged. The degree of neutrophil infiltration, which was determined by modifying the semiquantitative scoring method described previously, was graded from 0 to 3 (0 = no increase in the number of neutrophils; 1 = slight neutrophil infiltration; 2 = moderately dense neutrophil infiltration; and 3 = very dense neutrophil infiltration) [19].

### Assessment of gastric myeloperoxidase activity

Myeloperoxidase (MPO) activity was determined by following a previously published protocol [20]. In brief, tissue samples were weighed and suspended in a 50 mmol/l potassium phosphate buffer (pH 6.0) containing 5 mg/ml hexadecyltrimethylammonium bromide (Nacalai Tesque, Kyoto, Japan) at a ratio of 50 mg tissue to 1 ml of buffer. Tissues were homogenized, and 1 ml of the homogenate was decanted into sterile tubes and centrifuged at 12000 rpm for 15 min. Using a microtiter plate scanner, 200 μl of the reaction mixture, containing 16.7 mg of *o*-dianisidine (MP Biomedicals, Irvine, CA, USA), 90 ml of distilled H_2_O, 10 ml of potassium-phosphate buffer, and 50 μl of 1% H_2_O_2_, was added to each well, containing 20 μl of sample, in a standard 96-well plate, and 3 absorbance readings were recorded at 30-s intervals at 450 nm. MPO activity was measured in units/mg tissue, where 1 unit of MPO was defined as the amount needed to degrade 1 mmol of H_2_O_2_/min at room temperature.

### Quantitative analysis of gene expressions of proinflammatory cytokines and a chemokine

Total RNA was extracted from gastric tissue using the TRIzol reagent (Invitrogen, Carlsbad, CA, USA) and reverse transcribed using the Primescript 1st strand cDNA synthesis kit (Takara Bio, Otsu, Japan). Real-time polymerase chain reaction (PCR) was performed using the iQ SYBR Green Supermix kit (BioRad, Hercules, CA, USA) with the iCycler sequence detection system (BioRad). Briefly, 20 ng of cDNA was amplified at 95°C for 3 min and this was followed by 40 cycles of 95°C for 15 s and 60°C for 1 min using 10 μmol/l of gene-specific primers (IL-1β, IL-6, and CXCL1) and the iQ SYBR Green Supermix. Glyceraldehyde-3-phosphate dehydrogenase (GAPDH) was used as the housekeeping gene, and the ΔΔCT method was used to quantify the mRNA [21]. These primers were purchased from Takara Bio.

### Cell culture

A murine gastric mucosal cell line, GSM06, which was established from transgenic mice harboring the temperature-sensitive simian virus 40 large T-antigen gene and which can produce periodic acid-Schiff-positive glycoproteins, was obtained from Daiichi Pharmaceutical (Tokyo, Japan) [22]. The cells were cultured in Dulbecco’s modified Eagle medium/HamF12 medium (ICN Biomedicals, Irvine, CA, USA) containing 10% fetal bovine serum, 100 mg/ml streptomycin, and 100 IU/ml penicillin in 5% CO_2_ at 33°C as described previously [23].

### Lactate dehydrogenase release assay

Cell viability was assessed by a lactate dehydrogenase (LDH) release assay. LDH released from damaged cells was determined in aliquots of the culture medium according to the procedure described by the manufacturer (BioVision, Mountain View, CA, USA).

GSM06 cells were plated in a 96-well plate (2 × 10^4^ cells/well) and cultured. We determined the LDH release from GSM06 cells induced by 24-h treatment with 200 and 400 μM indomethacin after 1-h preincubation with or without 100 μg/ml sake yeast-derived TRX. As a reference, the dried material from normally treated Japanese sake (as described above), which contained very little TRX, was used. The absorbance at 490 nm was measured with a reference wavelength at 630 nm in the microplate reader. Experiments were performed in triplicate.

### Statistical analysis

All values were expressed as means ± standard error of the mean (SEM). When two groups were compared, the data were analyzed using Student’s *t*-test. When multiple groups were compared, the data were analyzed with one-way analysis of variance (ANOVA) followed by Fisher’s protected least significant difference test or the Kruskal–Wallis test followed by the Mann–Whitney *U*-test with Bonferroni correction, as appropriate, where possible using parametric or nonparametric analysis, respectively. A *P* value of less than 0.05 was accepted as statistically significant.

## Results

### Macroscopic findings of indomethacin-induced gastric mucosal injury

**Fig. 1:**
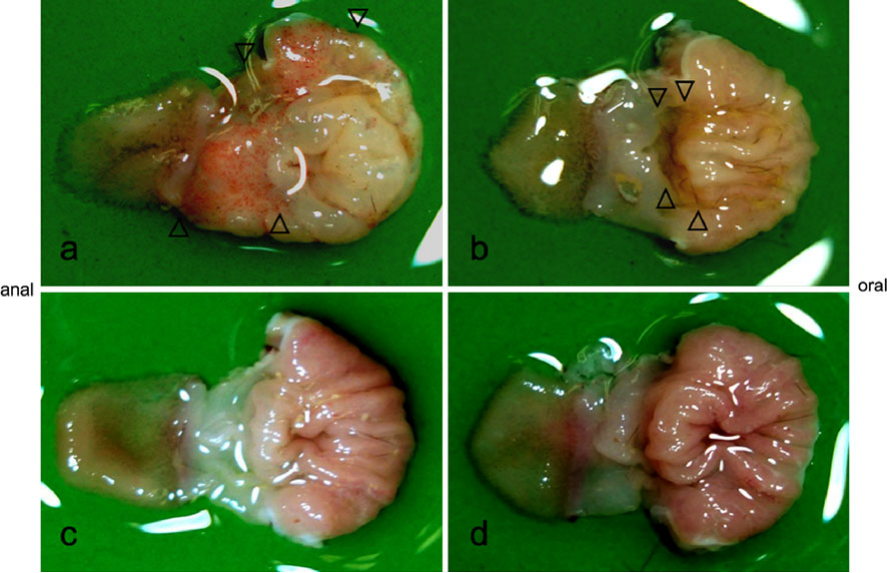
Representative macroscopic findings of the stomachs in Indomethacin-Control (**a**), Indomethacin-TRX (**b**), Saline-Control (**c**), and Saline-TRX (**d**) group mice. Indomethacin-Control group mice showed severely edematous gastric mucosae in the corpus and extensive hemorrhagic erosions or ulcers predominantly in the antrum (**a**). In the Indomethacin-TRX group mice (**b**), the extent of indomethacin-induced gastric mucosal injury was much smaller in size, and the severity of the injury was much milder than in the Indomethacin-Control group mice. Saline-Control (**c**) and Saline-TRX (**d**) group mice showed nonedematous normal gastric folds and intact mucosal surface. *Arrowheads* indicate the region of erosions or ulcers. *TRX* thioredoxin

Macroscopically, Indomethacin-Control group mice showed severely edematous gastric mucosae in the corpus and extensive hemorrhagic erosions or ulcers predominantly in the antrum (Fig. 1a). Saline-Control (Fig. 1c) and Saline-TRX (Fig. 1d) group mice showed nonedematous normal gastric folds and intact mucosal surfaces. The extent of the indomethacin-induced gastric mucosal injury in the Indomethacin-TRX group mice was much smaller in size and the severity was much milder compared with findings in the Indomethacin-Control group mice (Fig. 1b).

### Histological findings and assessments of indomethacin-induced gastric mucosal injury

**Fig. 2:**
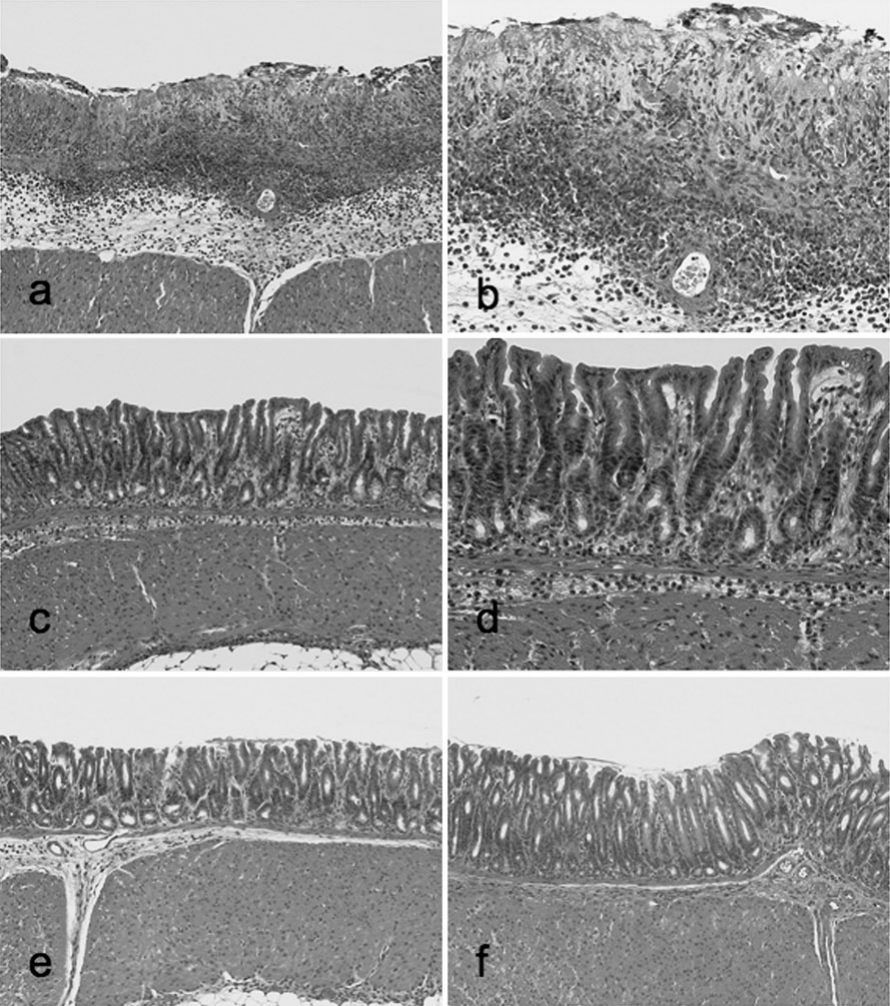
Representative histological findings of the antral mucosae in Indomethacin-Control (**a**, **b**), Indomethacin-TRX (**c**, **d**), Saline-Control (**e**), and Saline-TRX (**f**) group mice. Indomethacin-Control group mice exhibited typical findings of indomethacin-induced gastric mucosal injury (**a**, **b**): widespread necrosis with loss of surface epithelium, submucosal edema, and marked neutrophil infiltration. These findings were less apparent in Indomethacin-TRX group mice (**c**, **d**). Saline-Control (**e**) and Saline-TRX (**f**) group mice showed normal histological structure of the antral mucosae, regardless of the prophylactic administration of sake yeast-derived TRX. Original magnification: ×100 (**a**, **c**, **e**, **f**), ×200 (**b**, **d**), H&E staining (**a**–**f**)

Indomethacin-Control group mice exhibited typical findings of indomethacin-induced gastric mucosal injury predominantly in their antrum (Fig. 2a, b): widespread necrosis with loss of surface epithelium, submucosal edema, and marked neutrophil infiltration. These findings were less apparent in Indomethacin-TRX group mice (Fig. 2c, d). Saline-Control (Fig. 2e) and Saline-TRX (Fig. 2f) group mice showed normal histological structure of the stomach (antrum) regardless of the prophylactic administration of sake yeast-derived TRX.

**Fig. 3:**
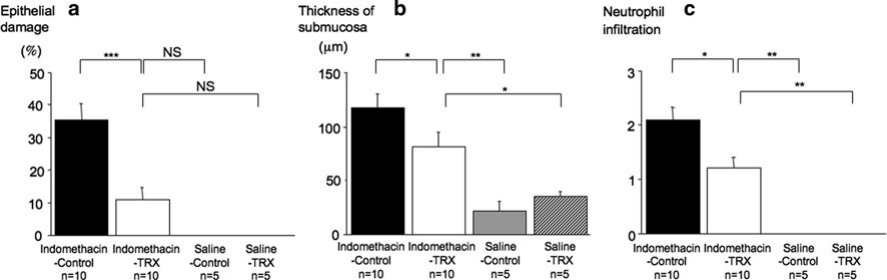
Epithelial damage (**a**), thickness of the submucosal layer (**b**), and degree of neutrophil infiltration (**c**) in Indomethacin-Control (*black bars*), Indomethacin-TRX (*white bars*), Saline-Control (*gray bar*), and Saline-TRX (*hatched bar*) group mice. There were significant differences in epithelial damage (**a**), thickness of the submucosal layer (**b**), and degree of neutrophil infiltration (**c**) in the antrum between Indomethacin-Control and Indomethacin-TRX group mice. In terms of epithelial damage, Indomethacin-TRX group mice showed no significant differences compared with Saline-Control and Saline-TRX group mice. In regard to the thickness of the submucosal layer and degree of neutrophil infiltration, Indomethacin-TRX group mice showed significant differences compared with Saline-Control and Saline-TRX group mice. Data are expressed as the means (±SEM) of 10 Indomethacin-Control, 10 Indomethacin-TRX, 5 Saline-Control, and 5 Saline-TRX group mice. *P < 0.05, **P < 0.01, and ***P < 0.001, by one-way analysis of variance followed by Fisher’s protected least significant difference test (**a**, **b**) or the Kruskal–Wallis test followed by the Mann–Whitney *U*-test with Bonferroni correction (**c**). *NS* not significant

There were significant differences in epithelial damage (Indomethacin-Control vs. Indomethacin-TRX: 35.0 ± 5.4 vs. 11.0 ± 3.6%, *P* < 0.001) (Fig. 3a), thickness of the submucosal layer (Indomethacin-Control vs. Indomethacin-TRX: 118.3 ± 12.1 vs. 82.0 ± 13.1 μm, *P* < 0.05) (Fig. 3b), and degree of neutrophil infiltration (Indomethacin-Control vs. Indomethacin-TRX: 2.1 ± 0.2 vs. 1.2 ± 0.2, *P* < 0.05) (Fig. 3c) in the antrum between Indomethacin-Control (*n* = 10) and Indomethacin-TRX (*n* = 10) group mice, as defined in “Methods”.

In terms of epithelial damage, Indomethacin-TRX group mice showed no significant difference compared with Saline-Control (0 ± 0%, *n* = 5) and Saline-TRX (0 ± 0%, *n* = 5) group mice. In regard to the thickness of the submucosal layer, Indomethacin-TRX group mice showed significant differences compared with Saline-Control (22.0 ± 8.9 μm, *n* = 5, *P* < 0.01) and Saline-TRX (34.9 ± 5.4 μm, *n* = 5, *P* < 0.05) group mice. In regard to the degree of neutrophil infiltration, Indomethacin-TRX group mice showed significant differences compared with Saline-Control (0 ± 0, *n* = 5, *P* < 0.01) and Saline-TRX (0 ± 0, *n* = 5, *P* < 0.01) group mice.

### Gastric MPO activity

**Fig. 4:**
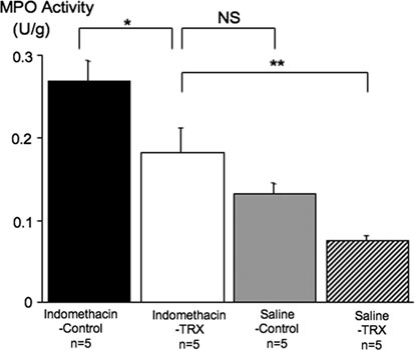
Gastric myeloperoxidase (*MPO*) activity in Indomethacin-Control (*black bar*), Indomethacin-TRX (*white bar*), Saline-Control (*gray bar*), and Saline-TRX (*hatched bar*) group mice. MPO activity in Indomethacin-Control group mice was significantly higher than that in Indomethacin-TRX group mice. Indomethacin-TRX group mice showed no significant difference compared with Saline-Control group mice, while they showed a significant difference compared with Saline-TRX group mice. Data are expressed as the means (±SEM) of 5 mice in each group. *P < 0.05 and **P < 0.01, by one-way analysis of variance followed by Fisher’s protected least significant difference test. *NS* not significant

Gastric MPO activity in Indomethacin-Control group mice (0.27 ± 0.03 unit/g tissue, *n* = 5) was significantly higher than that in Indomethacin-TRX group mice (0.18 ± 0.03 unit/g tissue, *n* = 5, *P* < 0.05) (Fig. 4).

Indomethacin-TRX group mice showed no significant difference compared with Saline-Control group mice (0.13 ± 0.01 unit/g tissue, *n* = 5), while they showed a significant difference compared with Saline-TRX group mice (0.08 ± 0.01 unit/g tissue, *n* = 5, *P* < 0.01).

### Gastric expressions of proinflammatory cytokine and chemokine genes by real-time PCR

**Fig. 5:**
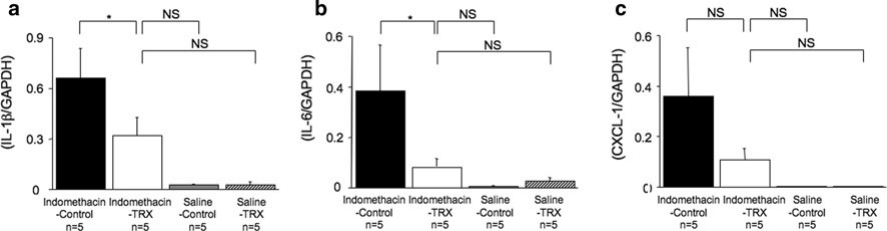
Gastric expression of proinflammatory cytokine and chemokine genes by real-time polymerase chain reaction (PCR) in Indomethacin-Control (*black bars*), Indomethacin-TRX (*white bars*), Saline-Control (*gray bars*), and Saline-TRX (*hatched bars*) group mice. Gastric mRNA expressions of interleukin-1β (*IL*-*1β*) and IL-6 in Indomethacin-Control group mice were significantly higher than those in Indomethacin-TRX group mice (**a**, **b**). Gastric mRNA expression of CXCL1 in Indomethacin-Control group mice tended to be higher than that in Indomethacin-TRX group mice (**c**). Indomethacin-TRX group mice showed no significant difference compared with Saline-Control and Saline-TRX group mice in gastric mRNA expressions of IL-1β, IL-6, and CXCL1 (**a**–**c**). Data are expressed as the means (±SEM) of 5 mice in each group. *P < 0.05, by one-way analysis of variance followed by Fisher’s protected least significant difference test. *NS* not significant. *GAPDH* glyceraldehyde-3-phosphate dehydrogenase

Gastric mRNA expressions of IL-1β and IL-6 in Indomethacin-Control group mice (IL-1β/GAPDH and IL-6/GAPDH: 0.66 ± 0.18 and 0.39 ± 0.18, *n* = 5) were significantly higher than those in Indomethacin-TRX group mice (IL-1β/GAPDH and IL-6/GAPDH: 0.32 ± 0.10 and 0.08 ± 0.03, *n* = 5, *P* < 0.05) (Fig. 5a, b). Gastric mRNA expression of CXCL1 in Indomethacin-Control group mice (CXCL1/GAPDH: 0.36 ± 0.19, *n* = 5) tended to be higher than that in Indomethacin-TRX group mice (CXCL1/GAPDH: 0.11 ± 0.04, *n* = 5, *P* = 0.09) (Fig. 5c).

Indomethacin-TRX group mice showed no significant difference compared with Saline-Control (IL-1β/GAPDH, IL-6/GAPDH, and CXCL1/GAPDH: 0.03 ± 0.01, 0.01 ± 0.00, and 0.00 ± 0.00, *n* = 5) and Saline-TRX (IL-1β/GAPDH, IL-6/GAPDH, and CXCL1/GAPDH: 0.03 ± 0.02, 0.03 ± 0.01, and 0.00 ± 0.00, *n* = 5) group mice in gastric mRNA expressions of IL-1β, IL-6, and CXCL1.

### LDH release assay to show the cytotoxic effect of indomethacin in GSM06 cells

**Fig. 6:**
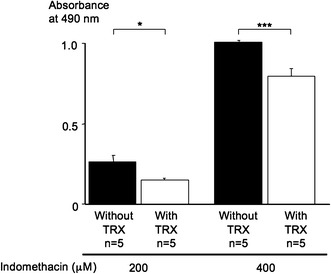
Lactate dehydrogenase (LDH) release from GSM06 cells induced by 24-h treatment with 200 and 400 μM indomethacin after 1-h preincubation with (*white bars*) or without (*black bars*) 100 μg/ml sake yeast-derived TRX (*n* = 5 in each group). The absorbance at 490 nm in the aliquots of the culture medium of GSM06 cells induced by treatment with 200 and 400 μM indomethacin after preincubation with 100 μg/ml sake yeast-derived TRX was significantly lower than that induced by preincubation without TRX. As a reference, the dried material from normally treated Japanese sake, which contained very little TRX, was used. Data are expressed as means (±SEM). Experiments were performed in triplicate. *P < 0.05 and ***P < 0.01, by Student’s *t*-test

LDH analysis showed that the absorbance at 490 nm in the aliquots of the culture medium of GSM06 cells induced by treatment with 200 and 400 μM indomethacin after preincubation with 100 μg/ml sake yeast-derived TRX (indomethacin 200 and 400 μM: 0.153 ± 0.007 and 0.793 ± 0.040, *n* = 5) was significantly lower than that induced by preincubation without TRX (indomethacin 200 and 400 μM: 0.261 ± 0.040 and 1.029 ± 0.004, *n* = 5, *P* < 0.05 and 0.001) (Fig. 6).

## Discussion

Previous studies have demonstrated that the overexpression of TRX in transgenic mice or the administration of recombinant human TRX attenuated a variety of stress-associated disorders [11–16]. TRX plays crucial roles in vivo as an antioxidant and a redox-regulating signaling molecule. In the present study, we have shown that indomethacin-induced gastric mucosal injury was dramatically attenuated in Indomethacin-TRX group mice, which were offered prophylactic oral administration of non-genetically modified sake yeast-derived TRX. Moreover, we observed a significant suppression of indomethacin-induced cytotoxicity (LDH release) by sake yeast-derived TRX in GSM06 cells. This was in line with a previous report that indomethacin-induced gastric injury was suppressed in human TRX transgenic mice [17].

In the stomach, PGs, especially PGE2, play an important role in maintaining gastric mucosal integrity via several mechanisms, including the regulation of gastric mucosal blood flow, turnover of epithelial cells, synthesis of mucus, and inhibition of gastric acid secretion [24]. Indomethacin is an NSAID that can suppress the production of PGs by inhibiting both COX1 and COX2. This, in turn, can lead to gastric mucosal damage as a side effect. Although indomethacin-induced gastric injury was significantly attenuated in TRX transgenic mice, Tan et al. [17] observed that PGE2 production was inhibited by indomethacin in TRX transgenic mice as well as wild-type mice, suggesting that the protective function of TRX against indomethacin-induced gastric injury is independent of the COX pathway.

It has been reported that indomethacin has pro-oxidant activity and initiates lipid peroxidation by generating ROS, which play a role in the gastric mucosal injury induced by indomethacin [25]. Previous studies have shown that TRX has radical scavenging activity against singlet oxygen and hydroxyl radicals [10]. Orally administered sake yeast-derived TRX might interact with membrane-bound TRX itself, or with a member of the TRX family on the cell surface, or it may enter the cells via lipid rafts to suppress intracellular ROS [26, 27]. In the present study, we showed a significant suppression of indomethacin-induced cytotoxicity by sake yeast-derived TRX in GSM06 cells, and Tan et al. [17] demonstrated that pretreatment with human TRX suppressed the indomethacin-induced generation of ROS in RGM-1 cells. Therefore, the suppression of indomethacin-induced gastric mucosal injury that we found in Indomethacin-TRX group mice may have been partly due to the suppression of ROS by TRX.

Wallace et al. [6, 28] reported that indomethacin-induced gastric injury was clearly dependent on neutrophils, and ROS production derived from activated neutrophils seems to play a crucial role in this injury. Previous studies showed that TRX inhibited neutrophil infiltration into inflammation sites in an air pouch model [29, 30]. In the present study, prophylactic oral administration of non-genetically modified sake yeast-derived TRX also significantly decreased the degree of neutrophil infiltration and MPO activity, a marker of neutrophil aggregation, in gastric tissue treated with indomethacin. These findings suggest that sake yeast-derived TRX suppresses ROS production in the tissue of indomethacin-induced gastric injury due to its inhibition of the infiltration of activated neutrophils into the mucosa. Furthermore, we found that sake yeast-derived TRX inhibited the production of proinflammatory cytokines (IL-1β, IL-6) and a chemokine (CXCL1) in the gastric mucosa and this agent attenuated neutrophil accumulation in the injured mucosa.

The administration of TRX suppresses oxidative stress-induced apoptosis [27]. Akt is a crucial signaling molecule with effects on cell survival and apoptosis. Akt has a redox-sensitive intramolecular disulfide bond between its Cys-297 and Cys-311 moieties and is redox-regulated. Hydrogen peroxide induces disulfide formation and inactivates Akt, and glutaredoxin, a redox-regulating protein, protects Akt from inactivation [31]. In addition, a previous study showed that TRX inhibited the indomethacin-induced inactivation of Akt and preserved the phosphorylation of Akt [17]. These lines of evidence suggest that TRX shows a protective effect against indomethacin-induced cytotoxicity at least partly through the preservation of the Akt-dependent antiapoptotic signal.

Clinically, there are many topics for discussion around NSAID-induced gastric injury. NSAID-induced gastric injury is greatly affected by *Helicobacter pylori* infection. In a previous study, Kawasaki et al. [14] demonstrated that the overexpression of TRX in transgenic mice and the administration of recombinant TRX attenuated *Helicobacter felis*-induced gastritis. Therefore, we can assume that sake yeast-derived TRX would also attenuate indomethacin-induced gastric injury concomitant with *Helicobacter* infection. NSAID-induced intestinal injury has pathological mechanisms similar to those of NSAID-induced gastric injury. In the present study we administered sake yeast-derived TRX orally, and TRX exerted effects on the gastric mucosa according to its high local concentration. However, oral administration of TRX will show much better efficacy against NSAID-induced gastric injury than against NSAID-induced intestinal injury, because TRX will have a lower concentration in the intestine. The long-term administration of low-dose aspirin (LDA) is associated with a high risk of adverse events, including LDA-induced gastric injury. However, aspirin and the other NSAIDs are different in their mechanisms of COX inhibition and gastric injury, and conclusions drawn from the damage induced by non-aspirin NSAIDs cannot necessarily be generalized to include aspirin-induced damage [32, 33]. Although the trigger mechanisms and the onset times of the gastric injuries induced by indomethacin and LDA are different, the gastric lesions induced by the agents are comparable, such as gastric erosions and ulcers. Therefore, TRX might have some good effect to reduce the mucosal injury induced by LDA. The use of a proton pump inhibitor (PPI) is the first-line treatment for NSAID-induced upper gastrointestinal injury at present. Evidence that acid exacerbates NSAID-induced injury provides a rationale for minimizing such damage by producing acid suppression [34]. Because the healing mechanisms of PPI and TRX against NSAID-induced gastric injury are completely different, the added healing effects of the two types of agents might represent a promising therapy for refractory NSAID-induced gastric injury with multiple risk factors.

As for the procedure of prophylactic oral administration of sake yeast-derived TRX, we referred to the previous literature [16] and intended to offer enough TRX to reduce gastric mucosal injury. Thus, we could show significant amelioration of indomethacin-induced gastric injury in mice. Potentially, we could have reduced the given dose and dosing period if we had performed a dose-finding study. As previously reported by other investigators, various functions of TRX have been proven to be dose-dependent in vitro [27, 30, 35, 36]. Judging from the cited literature, therefore, it is no wonder that TRX has a dose-dependent effect to reduce mucosal injury in vivo. Because there have been no TRX dose-finding studies in vivo (including the present study), we have nothing definite to state regarding the appropriate dose. In future investigations, we should confirm the appropriate given dose, dosing period, and dose-dependent effect of sake yeast-derived TRX in vivo.

In conclusion, the prophylactic oral administration of non-genetically modified sake yeast-derived TRX significantly inhibited the acute gastric mucosal injury induced by indomethacin in mice. This effect may be due, in part, to a reduction of neutrophil infiltration into the gastric mucosa, the inhibition of proinflammatory cytokine and/or chemokine production, and the suppression of intracellular ROS. Our results demonstrate, for the first time, that the oral administration of non-genetically modified sake yeast-derived TRX inhibits the pathogenesis of indomethacin-induced gastric injury. Prophylactic oral administration of sake yeast-derived TRX may be clinically useful to reduce NSAID-induced gastric mucosal damage.
